# Discovery and validation of candidate genes for grain iron and zinc metabolism in pearl millet [*Pennisetum glaucum* (L.) R. Br.]

**DOI:** 10.1038/s41598-020-73241-7

**Published:** 2020-10-06

**Authors:** Mahesh D. Mahendrakar, Maheshwari Parveda, P. B. Kavi Kishor, Rakesh K. Srivastava

**Affiliations:** 1 International Crops Research Institute for Semi-Arid Tropics (ICRISAT), Patancheru, Hyderabad, 502 324 India; 2 Department of Genetics, Osmania University (OU), Hyderabad, 500 007 India; 3 Department of Biotechnology, Vignan’s Foundation for Science, Technology and Research, Vadlamudi, Guntur, 522 213 India

**Keywords:** Genetics, Plant sciences, Genomics, Sequencing

## Abstract

Pearl millet is an important crop for alleviating micronutrient malnutrition through genomics-assisted breeding for grain Fe (GFeC) and Zn (GZnC) content. In this study, we identified candidate genes related to iron (Fe) and zinc (Zn) metabolism through gene expression analysis and correlated it with known QTL regions for GFeC/GZnC. From a total of 114 Fe and Zn metabolism-related genes that were selected from the related crop species, we studied 29 genes. Different developmental stages exhibited tissue and stage-specific expressions for Fe and Zn metabolism genes in parents contrasting for GFeC and GZnC. Results revealed that *PglZIP, PglNRAMP* and *PglFER* gene families were candidates for GFeC and GZnC. Ferritin-like gene, *PglFER1* may be the potential candidate gene for GFeC. Promoter analysis revealed Fe and Zn deficiency, hormone, metal-responsive, and salt-regulated elements. Genomic regions underlying GFeC and GZnC were validated by annotating major QTL regions for grain Fe and Zn. Interestingly, *PglZIP* and *PglNRAMP* gene families were found common with a previously reported linkage group 7 major QTL region for GFeC and GZnC. The study provides insights into the foundation for functional dissection of different Fe and Zn metabolism genes homologs and their subsequent use in pearl millet molecular breeding programs globally.

## Introduction

Nutritional security is the key component to improve the health status of the world’s population as they are primarily dependent on plant-based diets. Plants are the major source of essential nutrients and nutraceuticals for the normal growth and development of humans. However, half of the global population, especially people from Asia and Africa suffer from a deficiency of micronutrients such as Fe, Zn, and selenium as they depend upon cereal crops for food^1^. Biofortification is an approach to overcome the micronutrient deficiency/starvation by delivering nutrient-dense crops at the doorsteps of resource-poor people^2^. Biofortification under HarvestPlus-Consultative Group for International Agricultural Research (CGIAR) micronutrients project has focused primarily on seven major staple crops (rice, beans, cassava, maize, sweet potato, pearl millet, and wheat) targeting important micronutrients like Fe and Zn^3^. It is reported that 79% of pre-school and school-going children and 56% of women in India suffer from anemia due to iron deficiency^4^. Likewise, 50% of the world population suffers from diarrhea, impaired physical growth, and suppressed immune function due to zinc deficiency^5^. Fe supplementation program in India started in 1970 but failed to address the problem of iron deficiency^6^. To address malnutrition, high grain Fe and Zn containing pearl millet lines were developed using the biofortification approach of conventional breeding (HarvestPlus, 2009). Earlier reports have been revealed no significant correlation in grain yield and seed size with that of grain Fe and Zn content. Hence, while breeding for high mineral content in pearl millet, the large grain size was recommended for selection as an associated trait. The progenies derived from AIMP92901, a high-yielding OPV, exhibited large intra-population variability with the highest levels of Fe (~ 110 mg/kg) and Zn (~ 65 mg/kg) than their parents^7^.

An alternative approach to address the issue of genetic enhancement of grain Fe, Zn content is by modulating the metal transporters that facilitate their uptake, translocation, and storage^8^. Study of a gene family of regulated transporter-like protein (ZIP), Zn-regulated transporter and metabolism responsible for Fe and Zn homeostasis by either uptake or remobilization in intracellular compartments are crucial^9–11^. The ZIP transporters enhance Zn uptake in plant species like *Solanum lycopersicum*^12^, *Glycine max*^13^, *Arabidopsis thaliana*^14^, *Hordeum vulgare*^15^, and *Oryza sativa*^16^. ZIP is differentially regulated in various tissues under mineral deprivation and abundance in soils^17^. Millets and other cereals with high Fe and Zn content in seeds can be engineered by seed-specific expression of ZIP transporter genes. Fe and Zn are vital for plant growth, development and transport to the grain filling site^18^. As a structural motif, Zn plays an important role in many proteins, including DNA-binding Zn-finger proteins^19^, RING finger proteins, and LIM domain-containing proteins^19^, and participates in governing cellular processes such as growth, development, and differentiation. While sufficient Zn content enhances crop productivity^20^, Fe serves an important role in electron transfer during respiration and photosynthesis, though high concentrations of intracellular Fe may undergo Fe^3+^/Fe^2+^ redox reactions and cause damage to the plants^21^. Mineral deficiency hampers growth, yield and grain quality in cereals^22^, but excess Fe and Zn may cause significant toxicity to biological systems^23^. Therefore, plants developed a tightly regulated system to balance the uptake, utilization and storage of these metal ions^24^. Since Zn cannot diffuse across the cell membrane, specific Zn transporter is required to transport Zn into the cytosol and also into the stelar cells^25^. Metal transporters have been identified in plants, including the P_1B_-ATPase family, zinc-regulated transporter (ZRT), iron-regulated transporter (IRT)-like protein (ZIP), natural resistance-associated macrophage protein (NRAMP) family, and cation diffusion facilitator (CDF) family where these two metals play crucial roles^11^.

Several factors affect the uptake of both Fe and Zn from soil to roots, transport to leaves and stems and subsequently remobilization to developing grains^16^. Flag leaves are the major source of the remobilization of metals for developing seeds^26^. The gene(s) involved in Fe movement from soil to seed are not highly selective for Fe, and Zn, but other metals are also transported and allocated to different organs and extents^27^. Currently, it is not clear how many Fe and Zn metabolism genes are expressed in different tissues and at what developmental stages of pearl millet. This information is critical to understand the genetics and molecular mechanisms that help to identify candidate genes associated with their accumulation, toxicity, and homeostasis. Deployment of such information has been used recently in rice for developing lines with high grain Fe and Zn^28^. Several genes/gene families involved in Fe and Zn homeostasis like ZRT, ZIP, yellow stripe-like (YSL), NRAMP, nicotianamine synthase (NAS), nicotianamine aminotransferase (NAAT), heavy metal ATPases (HMA), metal tolerant protein (MTP)*,* zinc-induced facilitator-like (ZIFL) and others have been characterized in cereal crops. But a thorough understanding of the regulatory events leading to the roles and functions of Fe and Zn metabolism genes of pearl millet should help us to gain new insights into micronutrient homeostasis and ultimately mitigating malnutrition. Also, identifying their potential in metal transport, homeostasis, and their expression levels to regulate the ions is the prime aim of such an endeavor. The present study involves the identification of genes associated with Fe and Zn metabolism, gene structures, characterization, chromosomal localizations, grain Fe and Zn quantification and tissue-specific expression of these genes in *P. glaucum*. Unraveling the intricate mechanisms associated with Fe and Zn metabolism and the gene expression analysis should also help us in understanding better grain micronutrient nutrition in cereal crops^29^.

Pearl millet is a nutritionally important cereal not only for the rural poor but also in arid and semiarid tropics. Scientific research is still limited in comparison with other cereals^30^ in this regard. To date, no genes associated with Fe and Zn metabolism genes have been characterized in *P. glaucum* and also their transport capabilities at the time of grain filling. But, such findings help us in developing better lines with high grain Fe and Zn content and subsequently reducing malnutrition among resource-poor populations. As yet, a comprehensive analysis of all the Fe and Zn metabolism gene family members is not available in *P. glaucum*. An effort has been made to identify those genes that get expressed to mobilize nutrients from root to flag leaves and then into developing grains in pearl millet.

## Results

### In silico identification of Fe and Zn metabolism genes

A total of 114 Fe and Zn-responsive gene sequences from different plant species including *Setaria italica* (11), *Zea mays* (11), *Oryza sativa* (39), *Sorghum bicolor* (3) and *Arabidopsis* *thaliana* (49) were downloaded from NCBI. BLAST was performed against pearl millet sequences which resulted in the identification of 29 putative Fe and Zn metabolism genes. The reliability of these porters was checked by confirming the presence of Fe and Zn conserved domains. These genes were subdivided into five sub-families based on their conserved domains. The *YSL* was found to be a large family with 12 genes (with the exception of *PglYSL2* which was not considered due to a smaller length), followed by *ZIP* superfamily with 9 genes, *NRAMP* family with 6 genes and 1 each gene in *NAS* and ferritin-like (*FER*) families. For convenience, they are named as *PglYSL* for Yellow stripe-like proteins (*PglYSL1-12*), ZIP (*PglZIP1-9*), Nramp (*PglNRAMP1-6*), Ferritin like (*PglFER-1*) and NAS (*PglNAS-1*) (Supplementary Table S1).

### Chromosomal location and structure of Fe and Zn metabolism genes

**Figure 1:**
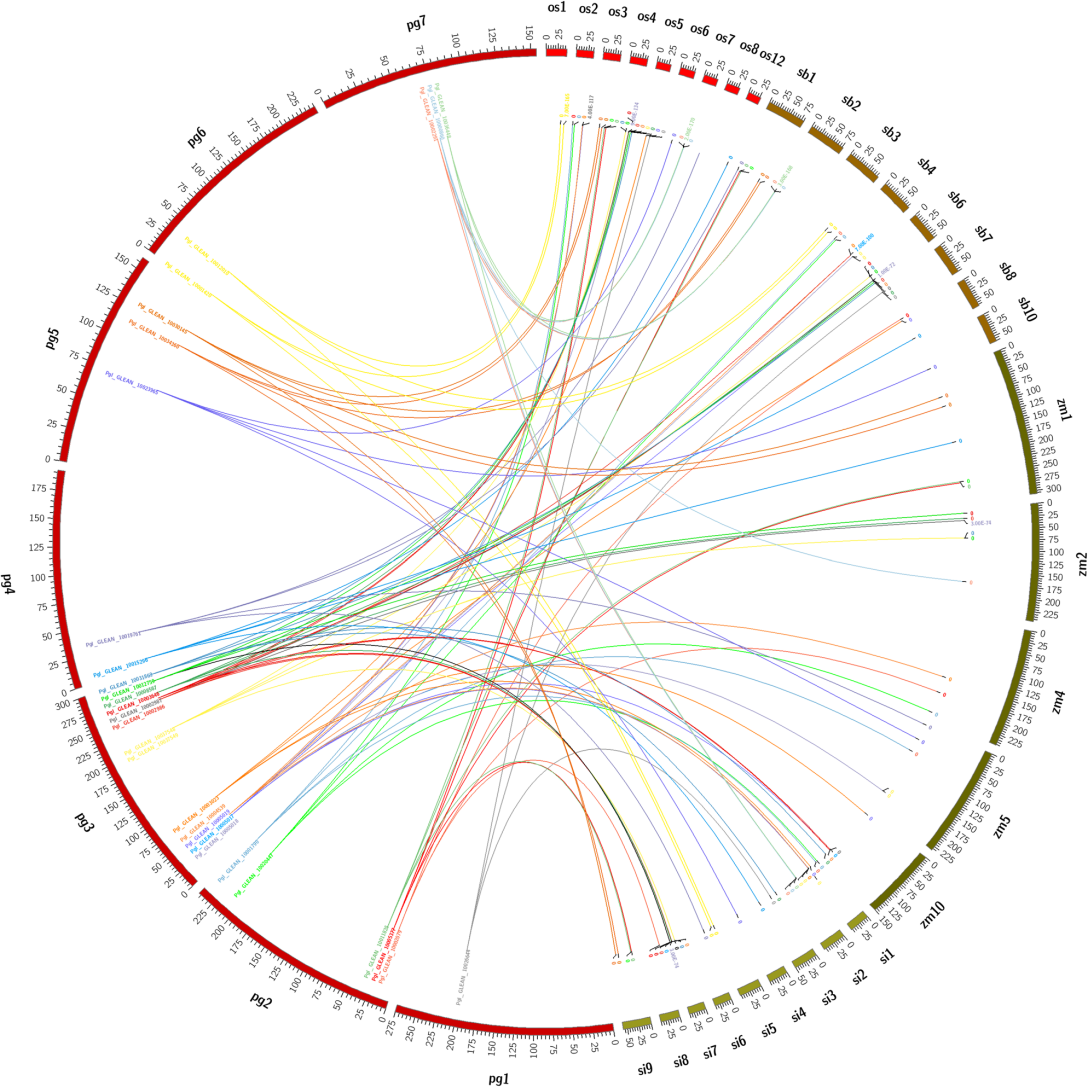
Circular genome visualization (CIRCOS) of pearl millet. Fe and Zn metabolism genes and their homologous map with other cereals (pink: *P. glaucum* chromosomes (Pg1–7); red: *O. sativa* chromosome (Os 1–12); brown: *S. bicolor* chromosomes (Sb 1–10); green: *Z. mays* chromosomes (Zm 1–10); light green: *S. italica* chromosomes (Si1–10).

**Figure 2:**
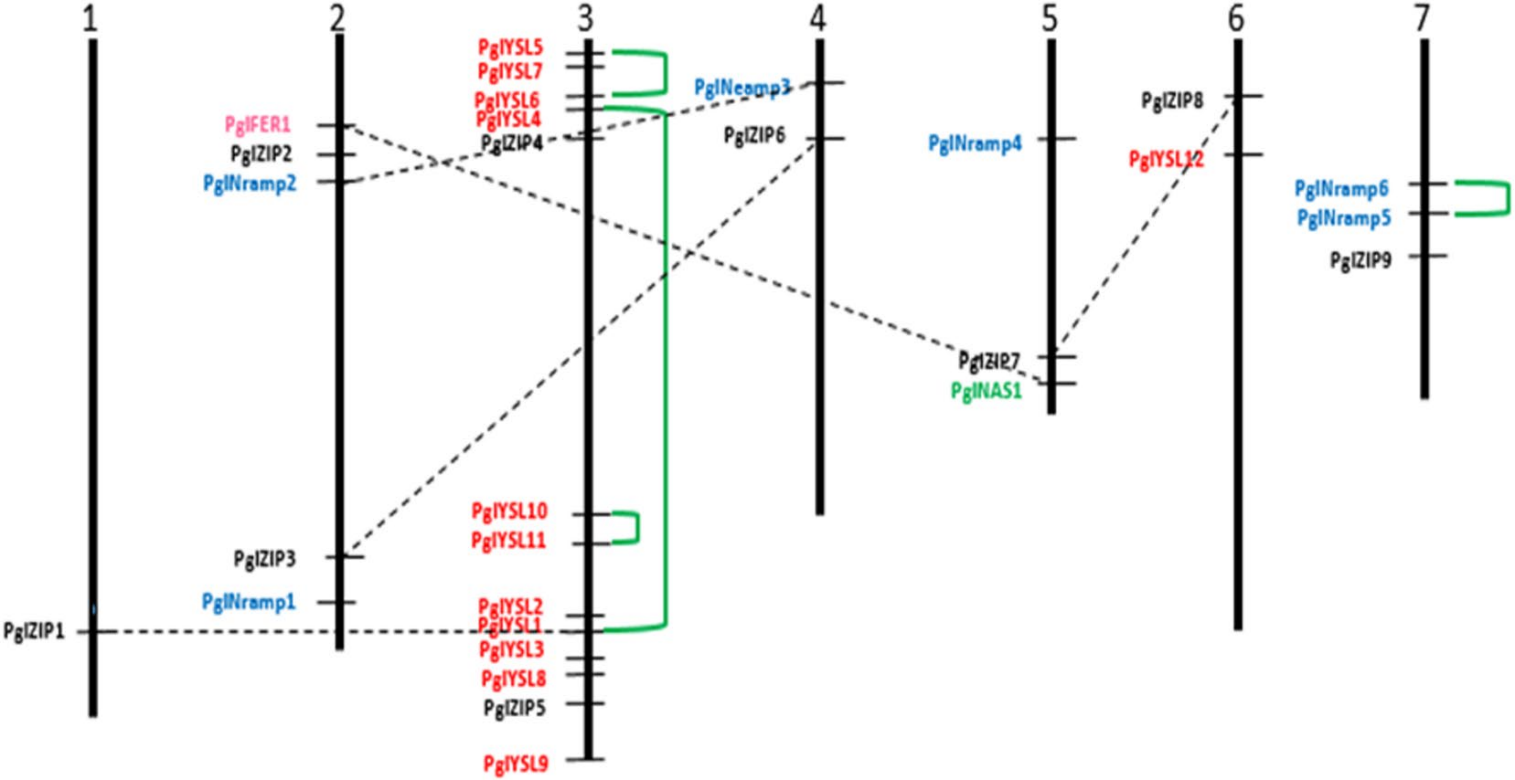
Chromosomal distribution of Fe and Zn metabolism genes in pearl millet identified through in silico mapping of LG7 high Fe and Zn QTL interval. Different families are illustrated by different colours and four tandem duplications are linked with dotted lines.

The 29 genes identified for Fe and Zn transporters in the present study were distributed on all 7 chromosomes of pearl millet and their homologs were found in *Setaria italica*, *Sorghum bicolor*, *Oryza sativa* and *Zea mays* which are shown in CIRCOS^31^ Fig. 1. Of the 29 genes, *PglZIP1* was found on chromosome 1; 5 genes on chromosome 2; 13 on 3, 2 on 4, 3 on 5, 2 on 6 and 3 on chromosome 7. Results indicated that chromosome 3 is the hot spot for these genes as it is homing maximum genes (Fig. 2). The gene structures analysis revealed that three genes are intron less while remaining displayed many introns. The exons ranged between 1 (Pgl_GLEAN_10002987) and 13 (Pgl_GLEAN_10000860) (Supplementary Table S1).

### Sequence analysis and conserved motif analysis of Fe and Zn metabolism genes

**Figure 3:**
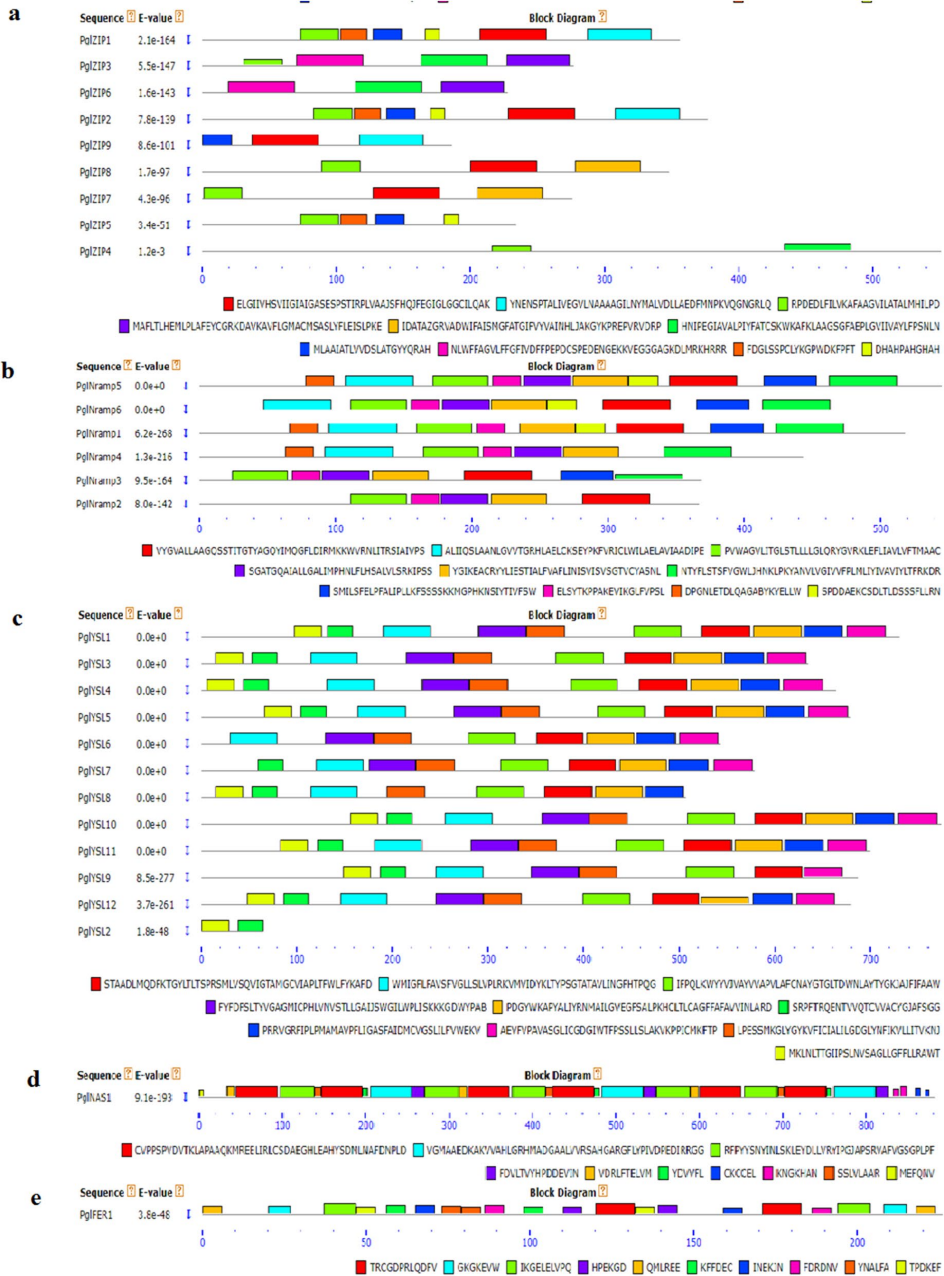
Conserved motif of Fe and Zn metabolism genes (**a**) 9 *PglZIP* motifs, (**b**) 6 *PglNRAMP* motifs, (**c**) 12 *PglYSL* motifs, (**d**) 1 *PglNAS* motif, (**e**) 1 *PglFER* motif. Gene clusters and p values are shown on the left side and motif sizes at the bottom of the figure.

The number of amino acids in the proteins ranged between 65 (*PglYSL2*, not considered as full-length gene) and 883 (*PglNAS1*); and the pI ranged from 5.32 (*PglFER1*) to 10.02 (*PgYSL2*). Out of 29 proteins, 10 were found acidic and the remaining basic. Molecular weights varied between 7146.53 (*PglYSL2*) and 96,605.68 (*PglNAS1*) daltons. Except for *PglFER1* and *PglNAS1,* all Fe and Zn transporter proteins were found hydrophobic. They showed the highest aliphatic index that varied from 132.59 (*PglNRAMP5*) to 82.52 (*PglFER1*). Transmembrane helices ranged from 1 to 15, but 2 proteins (*PglFER1* and *PglNAS1*) exhibited no helices. Subcellular localization revealed that of 29, 21 were in the plastid, 4 in the vacuole, 2 in the chloroplast, 1 each in the cytosol and extracellular region. The phosphorylation sites of these proteins (Supplementary Table S2) indicated that they are phosphorylated at serine residues, followed by threonine. But, the YSL subgroups were found phosphorylated at tyrosine residues. The PKC, CKII, PKA, UNSP, and CDC2 were common kinases for Fe and Zn protein phosphorylations. The identified Fe and Zn transporter proteins belonging to various subgroups did not display inter-subgroup similarity, hence each family was submitted to MEME separately for conserved motif analysis. The MEME analysis revealed ten conserved motifs, which showed various compositions and distribution patterns among the family members. All the YSL group proteins exhibited ten conserved motifs except YSL2, which showed 2 motifs. Motifs 6 and 10 were conserved at N terminus, while 5 and 8 at C terminus. Out of 29 identified genes, 10 showed conserved motifs among the YSL members. Motifs 6 and 3 are the most conserved at N terminus, while motifs 7 and 8 are commonly present at C terminus. In the ZIP family, motif 3 was found as the most conserved at N terminus, followed by motif 5, while 3 and 4 represent NES motifs. In the Nramp family, 10 were identified as the conserved motifs. While motif 3 and 9 were the most conserved at N terminus, motif 6 and 7 conserved at C terminus. Among all, motif 1 and 3 appeared as the utmost conserved (Fig. 3).

### Phylogenetic analysis

**Figure 4:**
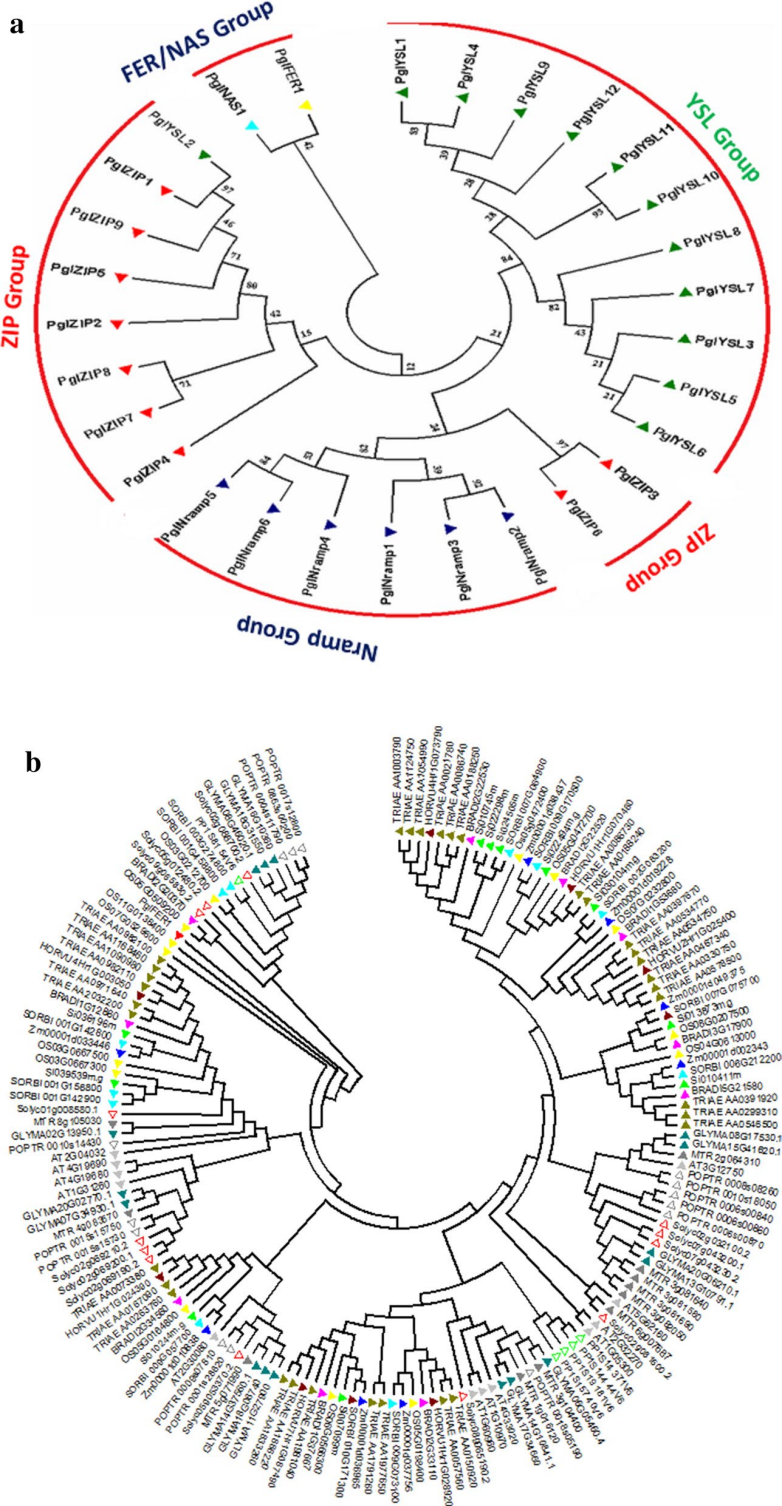

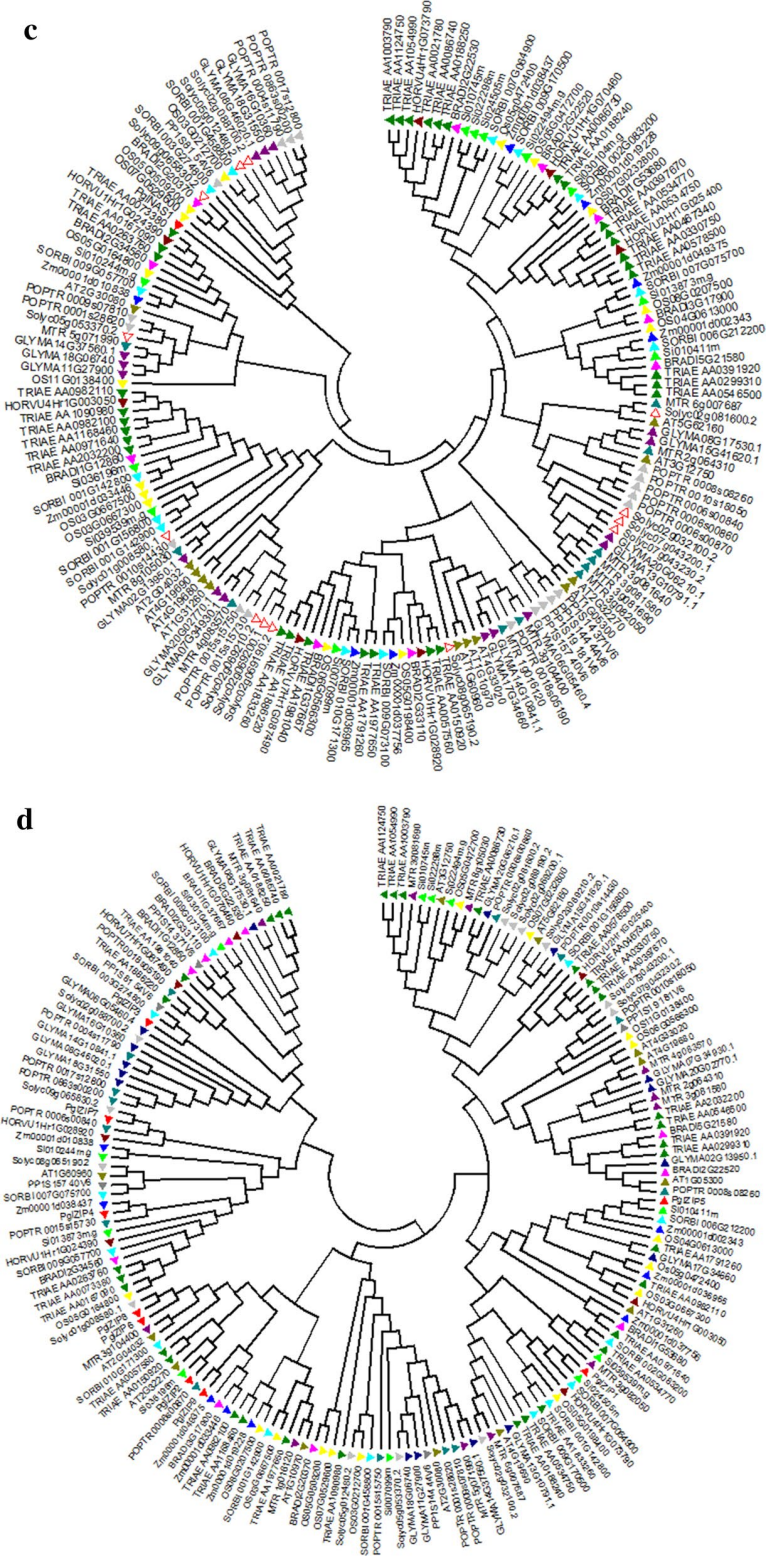

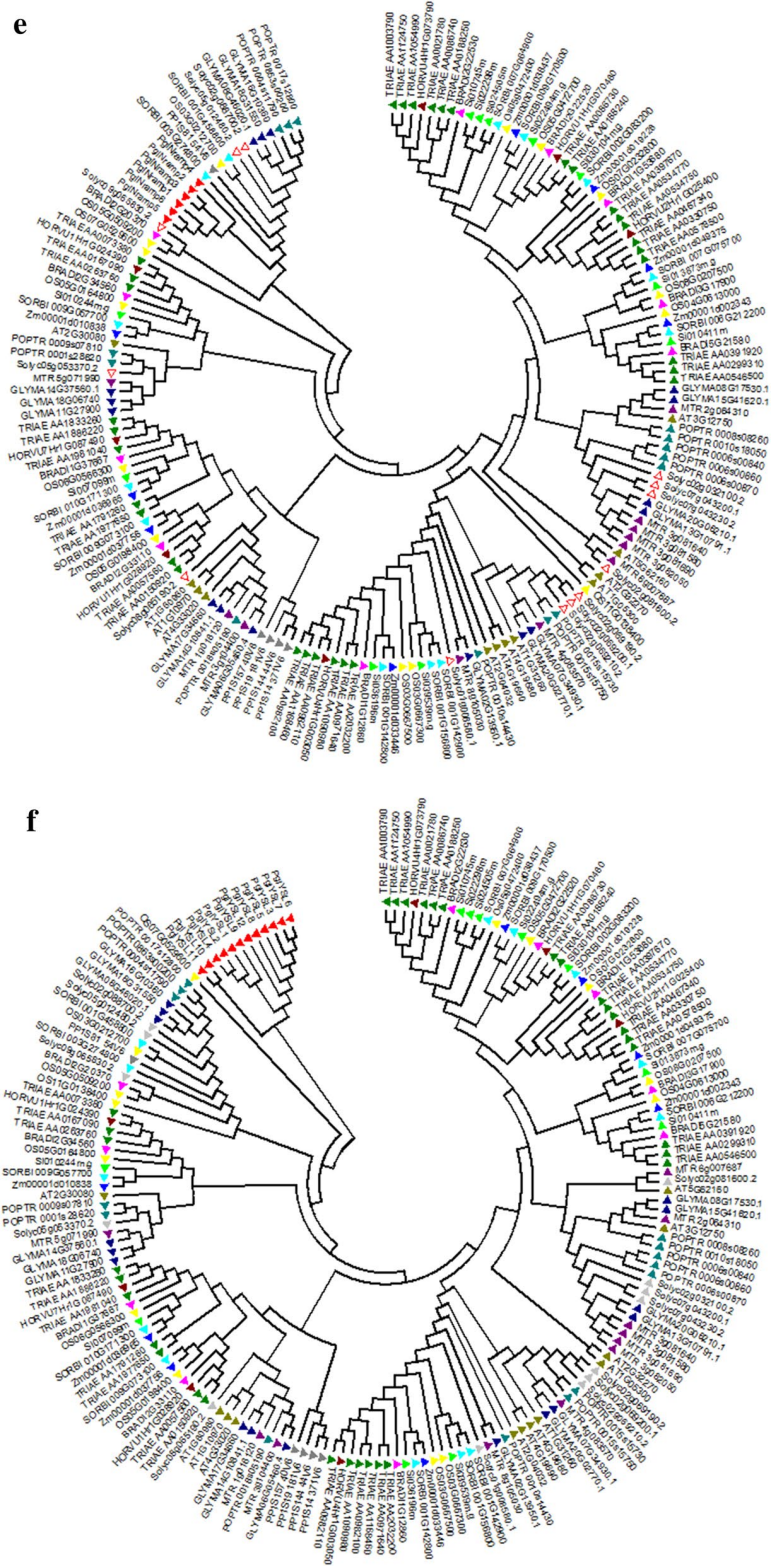
(**a**) Phylogenetic tree of Fe and Zn metabolic gene families in Pearl millet. N-J phylogenetic tree of Fe and Zn metabolism genes in pearl millet grouped into 4 showing ZIP, YSL, Nramp and FER. NAS is seen in a single group. A total of seven paralogs were observed out of which 4 segmental and 3 regional duplications were noticed. (**b**) Phylogenetic tree showing the relationship between Pearl millet, other cereals Ferritin (FER) family genes. (**c**) Phylogenetic tree showing the relationship between Pearl millet, other cereals NAS family genes. (**d**) Phylogenetic tree showing the relationship between Pearl millet, other cereals ZIP family genes. (**e**) Phylogenetic tree showing the relationship between Pearl millet, other cereals NRAMP family genes. (**f**) Phylogenetic tree showing the relationship between Pearl millet, other cereals YSL family genes.

A phylogenetic tree was constructed for 29 identified Fe and Zn metabolism genes, belonging to 5 groups in pearl millet. The analysis revealed four clades, of which five subfamilies clustered and showed group-specific distribution based on the conserved domains (Fig. 4a). While FER1 and NAS1 were clustered into one group, YSL2 fell into the ZIP family, ZIP3 and ZIP6 into the NRAMP subgroup. A total of 9 paralogs, 4 tandem duplications (*PglYSL1*/*PglYSL4*, *PglYSL5*/*PglYSL6*, and *PglYSL10*/*PglYSL11*) on chromosome 3, one *PglNRAMP5/PglNRAMP6* on chromosome 7, and 5 segmental duplication events were noticed. The phylogenetic tree was constructed to identify the evolutionary relationships of 5 gene families of Fe and Zn metabolism genes in pearl millet with other crop plants (Fig. 4b–f). The trees showed species-specific, group-specific and class-specific clades. Pearl millet showed 4 orthologous events, of which one with *Oryza* (*Os11G0138400/*Pgl_GLEAN_10003023), while remaining with *Setaria* (Si024505/Pgl_GLEAN_10026644, Si010411/Pgl_GLEAN_10012759 and Si03616/Pgl_GLEAN_1005377). All four genes belonged to the ZIP family (Fig. 4d).

### Promoter analysis

Promoter analysis revealed that the majority of them contain Zn deficiency-responsive (ZDRE), iron-responsive (IRO2) and iron deficiency elements (IDE), which are responsible for iron and zinc deficiencies. Interestingly, two IRO2 elements were found in *PglFER1* gene which is a potential candidate gene for GFeC. In *PglZIP2* and *PglZIP4,* single ZDRE element was found, whereas for *PglZIP2* two, *PglZIP4* one, *PglNRAMP5* two, and *PglZIP9* three IDEs were found. They also contained dehydration-responsive elements (DRE) for drought, low temperature-responsive elements (LTE), salt-regulated elements (SRE, GT1GM) and abscisic acid-responsive elements (ABRE). They exhibited the highest number of ABRE along with Cu-responsive elements. Among all, genes 3, 7, 11, 22, and 27 contained large amounts of stress-responsive elements (Supplementary Table S3).

### Grain Fe and Zn content

The grain Fe and Zn content in bi-parental RIL mapping population parents AIMP 92901-S1-183-2-2-B-08 (hereafter referred to as AIMP 92901) and ICMS 8511-S1-17-2-1-1-B-P03 (hereafter referred to as ICMS 8511) pearl millet lines were 110.70 ± 2.44 ppm, 79.11 ± 22.03 ppm, 31.26 ± 3.26 ppm and 26.63 ± 0.68 ppm, respectively. On the other hand, in control line MRC-HS-130-2-1-B-B-3-B-B-B-1-3-1 (hereafter called MRC), Fe and Zn content was 107 ± 14.22 ppm and 63 ± 13.03 ppm, respectively (Supplementary Table S4).

### Gene expression profiles in different tissues in contrasting genotypes

**Figure 5:**
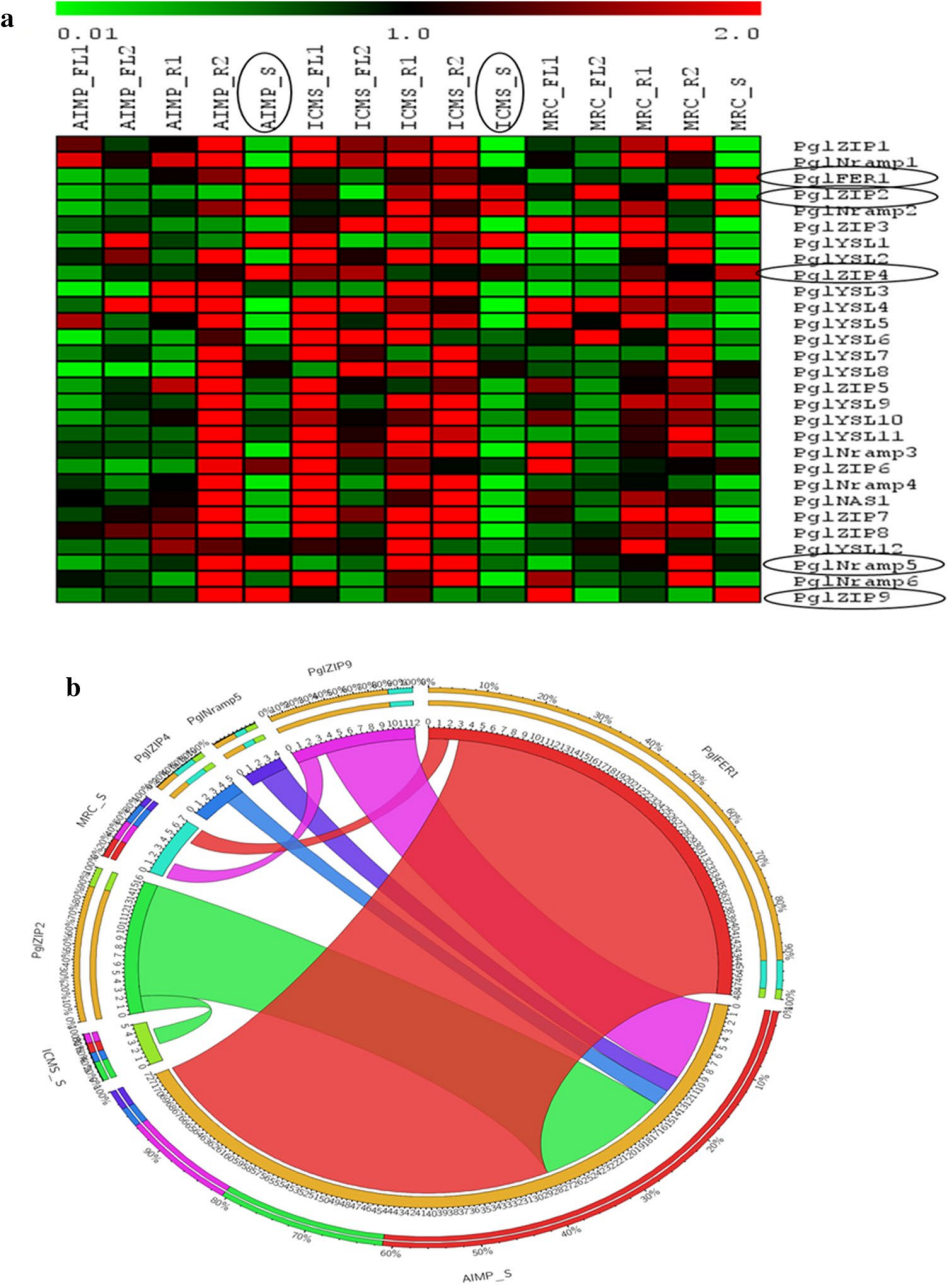
(**a**) Expression analysis of 29 Fe and Zn metabolism genes with five developmental stages of three genotypes (AIMP, ICMS and MRC). (**b**) Highly expressed genes shown in CIRCOS thickness of ribbon shows the expression levels.

To investigate the role of Fe and Zn metabolism genes in the accumulation of grain Fe and Zn content, the expression levels of genes were quantified by qRT-PCR analysis. The expression of these genes was analyzed among different tissue types and developmental stages to unravel their precise involvement in the transport, remobilization and grain loading of micronutrients. The expression values of different genes recorded during different developmental stages of three genotypes AIMP, ICMS and MRC are illustrated (Fig. 5a) by MultiExperiment viewer (MeV) v4.1.0. It has been noticed that the majority of the genes were up-regulated in root tissues at the reproductive stage compared to vegetative stages. In ICMS also, the up-regulation of genes was observed in roots and leaves at the vegetative stage. The *PglFER1, PglZIP2, PglZIP8, PglNRAMP2, PglYSL1* were found to be the candidate genes with high expressions in seeds showed in CIRCOS^31^ (Fig. 5b). They showed moderate expressions in the roots at the vegetative stage, indicating their Fe and Zn restoration nature in seeds. In ICMS, the majority of them were expressed at the vegetative stage compared to the reproductive stage, while in AIMP they were expressed in roots at the reproductive stage similar to MRC, the check.

### Correlation of differentially expressed genes with previously reported QTLs

The earlier identified LG1 and LG7 high grain QTL regions for GFeC and GZnC^7^ were scanned for presence of *PglZIP, PglNRAMP, PglYSL* and *PglFER* family genes that were differentially expressed between the two parents. Upon sequence comparison between these genes and the QTL regions defined by the set of linked markers, we could not locate any common set of genes for LG1 QTL interval. However, interestingly, for LG7 QTL interval two gene families, *PglZIP* and *PglNRAMP* were found to be common.

### miR159 associated with Fe

A host of plant miRNAs were screened in addition to checking the miRNAs from miRBASE. miR159 is the only miRNA that appeared to be associated with Fe, but, no miRNAs could be found for Zn. Surprisingly, careful dissemination and search from PMRD yielded only one miRNA in pearl millet. Before this, we have considered all the miRNAs in *Arabidopsis* and made a Smith-Waterman/Blast search to find any potential miRNAs, but in vain except miR159.

### Protein–protein interaction analysis predictions

**Figure 6:**
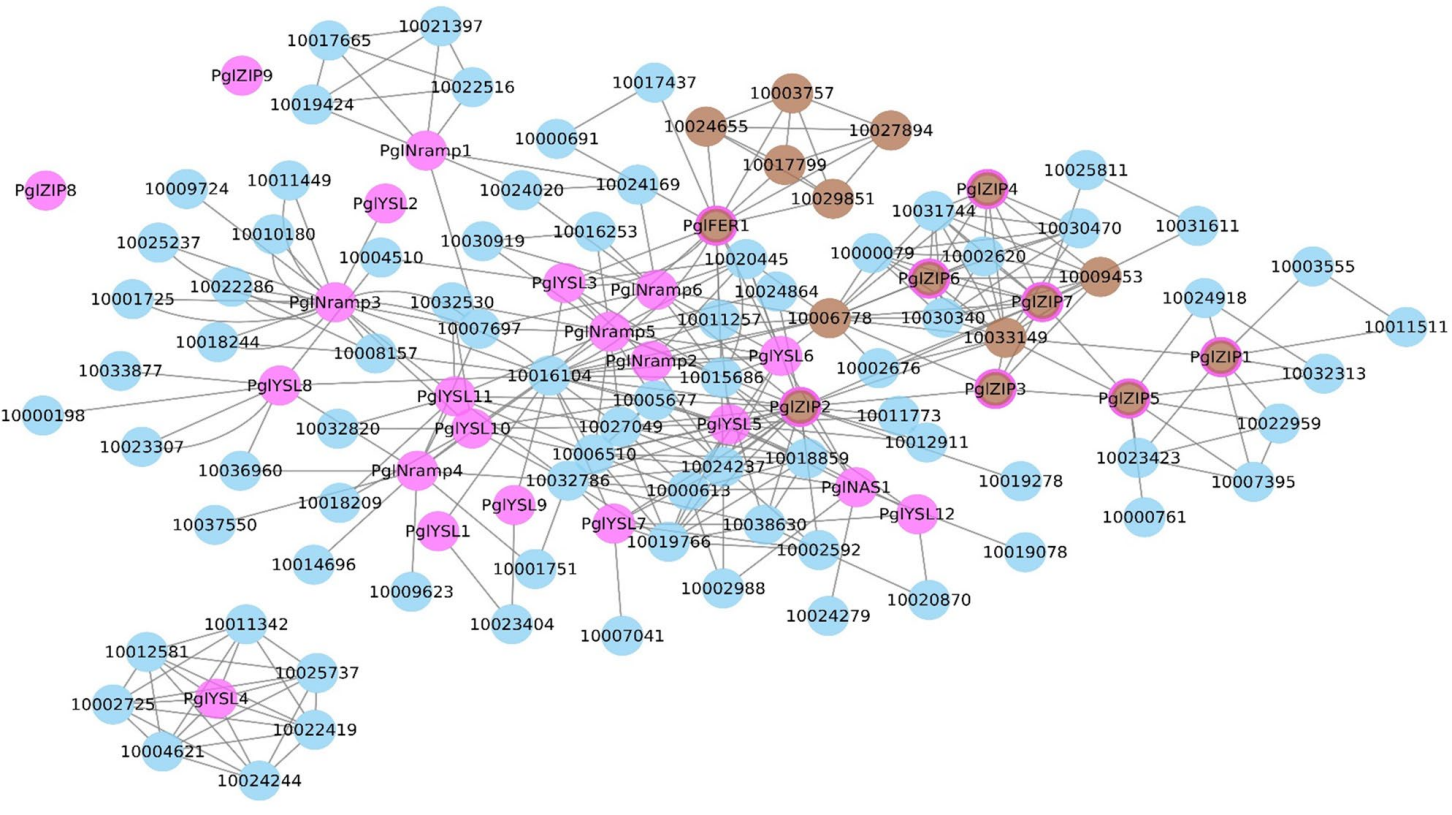
Protein–protein interaction (PPI) network for 29 proteins. Magenta nodes indicate 29 identified Fe and Zn metabolism proteins. Blue nodes indicate proteins interacting with 29 short listed proteins. Brown nodes indicate known proteins involved in the Fe and Zn transport. Brown with magenta border nodes indicate short listed proteins involved in Fe and Zn metabolism.

The predicted protein–protein interaction network revealed interactions of Fe and Zn accumulation associated proteins with several other transporter proteins of pearl millet (found ontology with *Sorghum* and *Seteria*). Fe and Zn transporter proteins have been noticed as centers for interactions and displayed interactions with various other proteins associated with the metabolism of fructose, galactose, and also with ATP binding protein, hexokinase and protein kinase (Supplementary Table S5; Fig. 6).

## Discussion

The deficiencies and improper consumption of micronutrients like minerals and vitamins lead to multiple disorders and diseases in humans and are collectively stated as hidden hunger or malnutrition. The connecting link between health and food has been well documented as human beings require several organic and mineral nutrients for their proper growth and development. Hyper and hypo levels of iron and zinc in the soil cause many physiological and metabolism-related complications in plants. But plants have developed a tightly regulated cellular iron and zinc homeostasis, including the uptake, distribution within different tissues and their utilization^32^. Since both iron and zinc are the vital nutrients, their deficiencies can lead to a variety of problems like retarded growth, skeletal abnormalities, learning capacity and wellbeing of humans^33^. Their deficiencies cause nutritional disorders in human beings, especially in developing world^34^. Edible parts of many cereal cultivars have inherently low iron and zinc content. Millets are nutritionally rich and can serve potentially as crops of nutritional security in the developing and underdeveloped countries. Therefore, biofortification of cereals or increasing their bioavailability in the edible parts like endosperm can cost-effectively solve the problem of malnutrition and save millions of lives across the globe^35^. Among the Fe and Zn superfamily, genes such as YSL, ZIP, NRAMP, FER, NAS have been identified in many plants like *Arabidopsis thaliana*^36^, *Setaria italica*^29^ and *Oryza sativa*^28^. These genes were responsible for Fe and Zn translocation from soil to the grain filling sight. Genome-wide studies of different families of Fe and Zn have been characterized individually in cereals, but there were no reports on the identification and gene expressions of Fe and Zn in pearl millet, though it has high micronutrients^37^. In the present study, 12 *PglYSL* , 9 *PglZIP*, 6 *PglNRAMP* and a single gene for *PglFER* and *PglNAS* were identified and characterized from *P*. *glaucum*. However, form *PglYSL* family, one gene *PglYSL*2 was found to be of much shorter length (just 65 amino acids). This may be because of differential divergence of this gene in pearl millet, or because of lack of capture of complete sequence during the genome assembly process. Hence, *PglYSL*2 was not considered in further studies.

Palmer et al.^38^ identified 520 genes that belong to 40 different transporter classes in the *Panicum virgatum* (C_4_ switchgrass) genome. There are five sub-families for Fe and Zn transport based on their conserved domains. Attempts were made earlier for in silico identification and expression analysis of either *YSL*^39^, or *ZIP*^40^ or *NAS* gene family^41^ or *NRAMP* members^42^ in a wide array of plants. The YS1 or YSL is a membrane-bound Fe (III)-phytosiderophores (PS) transporter^43^ and is distantly related to the Oligopeptide Transporter (OPT) family proteins. Though non-grass species do not synthesize PS, many YSL genes were detected in monocots, dicots, and lower groups of plants. Meena et al.^44^ identified 8 and 18 *YSL* family members in *Arabidopsis thaliana* and *Oryza sativa,* respectively. Similarly, in the present study, 12 genes belonging to the *YSL* family were detected suggesting that they are highly conserved across plant species. Species-specific variations in the gene numbers were also known to exist for many gene families in cereals^45^. The *ZIP* family members have been characterized in higher plants and are involved in metal uptake, transport, detoxification and storage of Fe and Zn in plant cells^46^. In *Oryza sativa*, 17 *ZIP* genes^47^, 15 in *Arabidopsis thaliana*^10^, 12 in *Poncirus trifoliata*^48^, and 9 in *Zea mays*^46^ were reported. Alagarasan et al*.*^29^ on the other hand, identified 36 ZIP family members in *Setaria italica*. In contrast, in the present study, 8 ZIP family members (out of a total of 29 iron and zinc transporter genes) were noticed in the genome of *P. glaucum*. This number is close to the published data in maize that belongs to the same family^46^. Members of the NRAMP family genes are also widespread from bacteria to plants to humans and transport Fe and Mn (manganese) across cellular membranes^49^. In the present study, 6 *NRAMP* members were identified in pearl millet like that of *Arabidopsis*^10^, while Qin et al*.*^42^ reported 13 in soybean. In *Arabidopsis*, 4 *FER* genes were detected^50^, and 3 in *Lupinus luteus*^51^. Gross et al*.*^52^ identified 2 *FER* genes in maize and rice, respectively, but only one is detected in the present study. A systematic analysis for NAS members could identify 3 and 4 NAS members in *Oryza sativa* and *Arabidopsis thaliana,* respectively^53^, while in the current study merely one NAS member was identified in pearl millet. But in maize and barley, 9 *NAS* genes were detected^41^. This suggests that NAS members in pearl millet are encoded by a few genes instead of a large gene family members. Zhou et al*.*^41^ pointed out that large NAS gene members in maize and barley may be due to gene duplication events that evolved during the evolution of new species.

YSL members are acidic with a large number of glutamic and aspartic acid residues. It was observed that these proteins are highly hydrophobic (27 out of 29) and are membrane-bound transporters. Except for two, the rest of the Fe and Zn porters have transmembrane helices in pearl millet. Such variability can occur between transmembrane domains 7 and 8 and also at the N-terminal portion of the YSLs. Chu et al*.*^39^ reported that *ysl1/ysl2* double mutants result in strong interveinal chlorosis and also reproductive defects implying that these two YSL members are needed for loading metals into the reproductive tissues. Menna et al*.*^44^ found that the intron/exon structure and domains of YSL members from rice and *Arabidopsis* are conserved during evolution. They also noticed less variation in sequence lengths of amino acid residues of rice (554–728) and *Arabidopsis* (664–724) YSL members. Though they are transport proteins, plasma membrane localization of these proteins could not be detected among the 29. But, others^44^ could detect plasma membrane-bound porters/proteins as the probable subcellular localization. This difference could be due to the diverse bioinformatics tool(s) that we use for this purpose. Guerinot^9^ pointed out that a metal-binding domain is seen between transmembrane 3 and 4. A phylogenetic tree was constructed to find out the evolutionary relationships of these porters. The *Arabidopsis* YSL family members share high sequence similarity. Based on sequence similarity, the YSL family can be divided into three conserved groups^39^, while we noticed only two groups (Fig. 3). It was noticed that amino acid sequences of *PglZIPs* were related to ZIPs identified in other plant species. This infers that *PglZIPs* share a common evolutionary ancestor along with other cereals. Two subfamilies appear for the members of Nramp that are common both in dicots and monocots as noticed in the present study and also in soybean^41^. Phylogenetic analysis revealed that NASs from the members of Poaceae were divided into two classes. Further, more members existed in class I in maize and barley than in rice^41^. Schnable et al*.*^54^ suggested that nearly 25% of the genes in maize possess closely related paralogs due to genome duplication events. Many *NAS* genes in some of the monocots indicate that there may be functional redundancy as also noticed by Mizuno et al*.*^55^. Nevertheless, the evidence is lacking at present to exclude or include the possibility of functional redundancy and if they are pseudogenes or not. Strozycki et al*.*^51^ working with *Lupinus* suggested that eudicot ferritins are both structurally and functionally diverse. They found out that 14-bp-long iron-dependent regulatory sequence (IDRS) is the characteristic feature for all higher plant ferritin promoters. They also found that a highly conserved IDRS can be extended (extIDRS) up to 22 bp. Such an analysis of ferritin gene promoters for specific or conservative elements and their validation only can resolve such a complexity. In the present study, the analysis of promoter regions was carried out for all the 29 genes. The presence of Fe and Zn deficiency elements, hormone-responsive, metal-responsive and salt-regulated elements indicate that Fe and Zn transporters are associated with diverse stresses. However, the promoters need to be validated further in the lab. The grain Fe and Zn content were higher in AIMP compared to ICMS indicating that AIMP should be utilized for future breeding programs. In pearl millet, QTLs for high GFeC and GZnC revealed a co-localization of QTLs on chromosome 3. Major QTLs were noticed earlier in other populations also, and LG3 and LG1 served as co-localizations for high grain Fe and Zn^56^. Current work thus forms a perfect computational framework for in silico characterization of five gene families that can be utilized in marker-assisted selection (MAS) of important cereal crops for enriching grain Fe and Zn.

The present work reveals that major differential gene expressions were recorded in *PglYSL2*, *PglZIP2, PglZIP8, PglNRAMP2, PglFER1* in the two contrasting parents. Such Fe and Zn transporter gene expressions in diverse tissues under various developmental stages provide evidence in the acquisition of iron and zinc, and tight regulation of metal ion homeostasis in the grains of cereals. Chu et al*.*^39^ demonstrated that *Arabidopsis* *YSL1* and *YSL3* are vital in iron transport and responsible for loading Fe, Cu, and Zn from source to sink i.e. grain filling site. On the other hand, YSL4 and YSL6 appear to be involved in iron transport and metal mobilization into the seeds of *Arabidopsis*^38^. Menna et al*.*^44^ reported a preferential pattern of YSL gene expressions in rice and *Arabidopsis* which suggest spatiotemporal regulation of YSL genes during plant development. Interestingly, both *AtYSL1*, *3* and *6* were highly expressed in senescent leaves indicating that they are implicated in the remobilization of metals^44^. It is known that some ZIP genes in *Arabidopsis* and rice play crucial roles in transporting Zn or Fe. ZIP genes exhibit different expression profiles like tissue specificity and response to the availability of Fe and Zn in the environment^57^. The gene expression patterns of *PglZIP*s indicate diverse functions carried out by ZIPs. While expression of *MtZIP1* was reported in Zn-deficient roots and leaves^58^, *MtZIP2* was stimulated in roots and stems by Zn deficiency^59^. Yang et al*.*^60^ observed that *OsZIP7a* was induced mostly in roots where Fe-deficiency is common, but *OsZIP8* was activated in Zn-deficient shoots as well as roots. In *Arabidopsis*, *bZIP19* and *bZIP23* regulated the adaptation to Zn deficiency by increasing the transcription of ZIPs^61^. In maize, *ZmZIP4*, *5*, *7* and *8* were upregulated in shoots and roots after excess Fe was applied^46^. They pointed out that *ZmZIP4*, *5*, *7* and *8* may be associated with excessive Fe detoxification and storage. Also, *ZmZIP4* and *ZmZIP5* were stimulated during embryo development and they were repressed in later stages^46^. Such an expression suggests that these two ZIPs are essential for the growth of plumule and radicle in maize. Qin et al*.*^42^ studied the soybean *NRAMP* gene expressions and suggested these genes function in many tissues and developmental stages. *GmNRAMP* genes were found to be differentially regulated upon N, P, K, Fe and S deficiencies as well as toxicities of Fe, Cu, Cd, and Mn. These results infer that *NRAMPs* are functionally associated with many nutrient stress-responsive pathways. Zhou et al*.*^41^ found that class I *NAS* genes were induced under Fe deficiency, but suppressed under excess Fe conditions in *Zea mays*. In contrast, they noticed that the expression pattern of class II *NAS* genes was a reversal of class I genes, thus complementing each other. While *ZmNAS1*; *1/1*; *2* was mainly expressed in cortex and stele of root tissues under sufficient Fe conditions, but under deficit conditions, its expression was noticed also in the epidermis, and shoot apices. The expression patterns suggest that the two classes may be regulated at the transcriptional level and respond to the demand for iron uptake and transport^40^. Fobis-Loisy et al*.*^62^ noticed in maize differential accumulation of FM1 and FM2 (two sub-classes) mRNAs in response to iron, ABA and water deficit conditions which could be due to differential transcription of *ZmFer1* and *ZmFer2*. Bournier et al*.*^63^ found out that a molecular link exists between the control of iron and phosphate homeostasis in *Arabidopsis*. Further, genome-wide analysis of gene expression profiling revealed that an intact COP9 signalosome is essential for correct expression of Fe homeostasis genes in *Arabidopsis*^64^. Such a possibility cannot be ruled out in pearl millet. Our *in silco* analysis has further validated key genes that are differentially expressed like ZIP, and NRAMP family members which have been found significantly on LG 7 QTL by annotation study and found to be a candidate gene.

Besides, miRBASE, other plant miRNAs were screened in the present study for identifying miRNAs associated with the regulation of Fe, Zn transporters^65^. We found that pgl-miR159 is known to be the only matured miRNA sequence that was identified from the Plant miRNA database (PMRD)^66^. With the plant miRNAs predominantly targeting transcription factors, we aimed to identify functional targeted genes responsible for various physiological processes using psRNAtarget^67^. For the lone miRNA, we found that 1-amino-cyclopropane-1-carboxylate synthase 8 (ACS8) is the probable target. While this gene was found in *Arabidopsis*, it is not yet annotated in pearl millet. We believe that ACS transcripts are responsible for Fe deficiency, which is invariably associated with increased abundance of ACS transcripts in plant leaves^68^. Our study revealed that among the 29 proteins, 6 proteins (*PglFER1, PglZIP2, PglZIP3, PglZIP4, PglZIP5,* and *PglZIP9*) formed tight networking with other proteins (Fig. 6). The protein Pgl_GLEAN_10005377 (*PglZIP2*) displayed the highest interactions. (Supplementary Table S5). *PglFER and PglZIP* might play a key role in mineral transportation and accumulation in seeds^69^.

## Conclusions

Pearl millet is a climate-resilient nutricereal with substantial genetic variability for grain Fe and Zn content. Our results from the gene expression studies, corroborated by QTL interval annotation revealed that the gene families *PglYSL, PglZIP, PglNRAMP, PglNAS,* and *PglFER* play an important role in Fe and Zn homeostasis in pearl millet. Ferritin-like gene *PglFER1* along with other key candidate genes should be further investigated in diverse pearl millet germplasm. This study provides insights into the foundation for functional dissection of different Fe, Zn metabolism genes homologs. The genomic resources developed in this study may be used for the development of breeder-friendly marker systems for improvement of grain Fe and Zn content in the pearl millet breeding programs globally.

## Materials and methods

### In silico identification of Fe and Zn metabolism genes

The Fe and Zn transporter gene sequences of *Setaria italica*, *Zea mays, Oryza sativa*, *Brachypodium distachyon,* and *Triticum aestivum* were retrieved from NCBI (https://www.ncbi.nlm.nih.gov/) database and searched against *Cenchrus americanus* (pearl millet) genome^70^ to find out their homologs. GENSCAN (http:// genes.mit.edu/GENSCAN.html) was used to retrieve the gene and corresponding protein sequences. All the identified putative protein sequences were subjected to Motif Search (https://www.genome.jp/tools/motif/) to check their reliability and identification of conserved domains^71^.

### Chromosomal locations and gene structure

The identified Fe and Zn metabolism genes are mapped to their respective chromosomes based on the information provided in the pearl millet genome database (http:// https://www.gigadb.org/dataset/100192). The gene structure display server (https://gsds.cbi.pku.edu.cn) software was used for obtaining the Fe and Zn gene structures—exons, introns, and untranslated sequence regions (UTRs) based on the alignments of their coding sequences. MEME software^72^ was employed to analyze the new sequence patterns, their distributions, significance^73^ and identify the motifs by setting different default parameters like the number of motifs from 1–10, with a motif width of 5–50, and the number of motif sites from 5–10.

### In silico protein analysis

The molecular weight (MW), isoelectric point (pI), and grand average of hydropathy (GRAVY) of Fe and Zn metabolism genes were identified using ProtParam of Expasy tools^74^ (https://web.expasy.org/protparam), while the phosphorylation sites were predicted by employing NetPhosK3 software of Expasy tools^75^. The putative transmembrane helices within genes were identified using TMHMM software^76^. For finding out the subcellular localization of transporters, the WOLFPSORT program (https://wolfpsort.org/) was used^77^.

### In silico prediction of *cis*-regulatory elements and phylogenetic analysis

Genomic sequences measuring 2000 bp of upstream of the start codon of Fe and Zn metabolism genes were extracted for the identification of *cis*-acting elements. The elements were analyzed by employing PLANTCARE software^78^. The Neighbor-Joining (NJ) phylogenetic tree was constructed for the Fe and Zn protein sequences of pearl millet and other cereals like *Setaria, Oryza,* etc., by using MEGA 6.2 software^79^ by employing the Poisson correction, pairwise deletion, and bootstrap value (1000 replicates) permutation.

### Plant material

In the present investigation, two contrasting parents of a bi-parental RIL mapping population AIMP 92,901 (high grain Fe and Zn), and ICMS 8511 (low grain Fe and Zn) were used^7^. Another line MRC was used as a check which records very high grain Fe and Zn content. The experiment was conducted under controlled environmental research facility (CERF), ICRISAT greenhouse conditions at the geographical location of longitude 17.51017° N, latitude 78.27540° E and altitude 623.14 m. Since the impact of the geographical location of plants remains a potential aspect while considering for nutrient accumulation and biological activities, the precise location of crop grown area is mentioned here. Following plant tissues were used for qRT-PCR analysis: flag leaf 1 (during vegetative stage), flag leaf 2 (during reproductive stage), root 1 (during active vegetative growth), root 2 (during the grain filling stage) and mature seeds.

### Element analysis for grain Fe and Zn content

Grain Fe and Zn content were analyzed at the Charles Renard Analytical Laboratory, ICRISAT, Patancheru, Hyderabad, India following the method described by Wheal et al*.*^80^. The ground samples were digested in closed tubes, and Fe and Zn in the digests were analyzed using Inductively Coupled Plasma Optical Emission Spectrometry (ICP-OES). RNA was isolated at the vegetative and reproductive stages. The flag leaf and root were harvested first at the vegetative stage once and again the same was collected at the mature embryo (reproductive) stage and frozen in liquid nitrogen and stored at − 80 °C until further use. The values are represented as means ± standard errors of the replicated data.

### RNA isolation, cDNA synthesis and qRT-PCR analysis

Total RNA was extracted from different tissues of pearl millet using the Invitrogen RNA isolation kit according to the manufacturer's instructions. A total of 2.5 μl RNA with 5 μg concentration was taken for conversion to cDNA using a first-strand cDNA synthesis kit (Thermo Scientific) for qRT-PCR analysis of pearl millet Fe and Zn transporters. The template was diluted with nuclease-free water (1:10). Gene expression analysis was performed for 29 gene-specific primers, along with three reference genes (Supplementary Table S6), namely elongation factor 1-alpha (EF-1α), eukaryotic initiation factor 4A (EIF4A) and an acyl carrier protein (ACP)^81^ for gene normalization in 96-well optical PCR plates. The SYBR Green PCR master mix (Applied Biosystems, CA, USA) was used for gene expression analysis according to the manufacturer’s instructions. The ABI 7500 real-time PCR system (Applied Biosystems, USA) was used with the following thermal cycling conditions; the cycle 1 at 95 °C for 10 min, followed by 40 cycles at 95 °C for 15 s and 60 °C for 1 min (variable based on primers gradient temperatures) (Supplementary Table S6). The amplicon dissociation curves were recorded with a fluorescent lamp after the 40^th^ cycle by heating from 58 to 95 °C within 20 min. Three biological replicates were taken in addition to two technical replicates each time. The relative expression values of 29 genes in five tissues as mentioned earlier at two developmental stages (vegetative and reproductive) of 3 contrasting genotypes (low and high Fe and Zn content) were calculated using qbase^+^ software^82^.

### Correlation of differentially expressed genes with previously reported QTLs

We correlated differentially expressed genes in the plant materials (ICMS 8511 and AIMP 92901) which also happen to be parents of a bi-parental RIL mapping population. This RIL population was earlier used to identity LG1 and LG7 high grain QTL regions for GFeC and GZnC^7^. Using in silico approaches, the LG1 and LG7 QTL interval was scanned for presence of common genes that were differentially expressed and were also present in the QTL region.

### In silico identification of miRNA(s)

For the in-silico identification of miRNA associated with Fe and Zn we have used 29 putative gene sequences as input to find perfect or complementary mi RNA binding to its target sequences. The pearl millet miRNA targets were identified through pairwise homolog search by subjecting mature miRNA sequences as query against manually curated reference mRNA sequences (RefSeq, NCBI) and assembled EST sequences of pearl millet using Plant small RNA Target^67^ (psRNATarget). Analysis server (https://plantgrn.noble.org/psRNATarget/) of Dai and Zhao^83^. The parameters suggested by Vishwakarma and Jadeja^84^, were deployed for prediction of miRNA targets in pearl millet. Further screened by using miRBASE and Plant miRNA database^66^ (PMRD): https://bioinformatics.cau.edu.cn/PMRD/. To further confirm this, we have also done a UVA Fasta/Smith-Watermann algorithm search using the Pearl millet and Arabidopsis whole genomic sequences taking the matured sequences as query from miRNA databases. The brief methodology representing in pictorial form in supplementary Fig. 1.

### Protein–protein interaction

The protein–protein interaction (PPI) was prepared using 29 identified genes. As the pearl millet PPI network details are not available in the STRING database, initially all the orthologs of *P. glaucum* in the *S. italica* were listed out. For each gene (29), corresponding and interacting proteins were listed by searching in the STRING database and redundant proteins were discarded. Network analysis and functional enrichment in the network were performed by Cytoscape^85^.

## Supplementary information

Supplementary file 1

Supplementary file 2

Supplementary file 3

Supplementary file 4

Supplementary file 5

Supplementary file 6

Supplementary file 7

## Data Availability

All the data presented in the manuscript are publicly available. The gene expression data generated during this study are available from the corresponding author(s) on request. All authors have read and accepted the MS.
