# Supplementary material for: Discovery and validation of candidate genes for grain iron and zinc metabolism in pearl millet [Pennisetum glaucum (L.) R. Br.]
Source: Sci Rep. 2020 Oct 6;10:16562. doi: 10.1038/s41598-020-73241-7 (PMC7538586; doi:10.1038/s41598-020-73241-7)
Supplement: Supplementary file 1 [file 41598_2020_73241_MOESM1_ESM.docx]

**Discovery and validation of candidate genes for high grain iron and zinc metabolism in pearl millet [*Pennisetum glaucum* (L.) R. Br.]**

**Mahesh D. Mahendrakar^1,2^, Maheshwari Parveda^2^, P. B. Kavi Kishor^2,3*^, Rakesh K. Srivastava^1*^**

^1^International Crops Research Institute for Semi-Arid Tropics (ICRISAT), Patancheru, Hyderabad - 502 324, India

^2^Department of Genetics, Osmania University (OU), Hyderabad - 500 007, India

^3^Department of Biotechnology, Vignan’s Foundation for Science, Technology & Research Deemed to be University, Vadlamudi, Guntur-522 213, India

Correspondence: [r.k.srivastava@cgiar.org](mailto:r.k.srivastava@cgiar.org), pbkavi@yahoo.com

**Supplementary Table 1**: List of Fe and Zn metabolism genes, and their chromosome localization, length of amino acid, CDS, pI/Mw, DBD,

Transmembrane helices, number of exons, NLS (nuclear localized sequence) and NES (nuclear export signal) and their sub-cellular localizations.

| **S.No.** | **Gene** | **Common name** | **Gene name** | **Chromosome location** | **No. of Amino acids** | **CDS** | **DBD(DNA Binding Domains** | **TMHMM** | **Intron** | **pI/MW** | **Instability index** | **GRAVY** | **Aliphatic index** | **localization** | **NES** |
| --- | --- | --- | --- | --- | --- | --- | --- | --- | --- | --- | --- | --- | --- | --- | --- |
| 1 | Pgl_GLEAN_10026644 | PglZIP1 | Zinc Transporter | 1 | 356 | 1071 | 43-353 | 9 | 2 | 6.04/36975.30 | 26.31 | 0.72 | 118.43 | V | 10 (LCLLVTV) |
|  |  |  |  |  |  |  |  |  |  |  |  |  |  |  |  |
| 2 | Pgl_GLEAN_10001790 | PglNramp1 | Nramp (Metal Transporter 9 like) | 2 | 518 | 1557 | 71-250 | 10 | 11 | 6.41/56317.12 | 32.08 | 0.592 | 115.21 | P | - |
|  |  |  |  |  |  |  |  |  |  |  |  |  |  |  |  |
| 3 | Pgl_GLEAN_10002079 | PglFER1 | Ferritin | 2 | 226 | 681 | 63-208 | 0 | 8 | 5.32/25355.70 | 35.93 | -0.372 | 82.52 | Chl | 154 (LALEKLVNE) |
| 4 | Pgl_GLEAN_10005377 | PglZIP2 | ZIP Fe (2+) Transporter | 2 | 377 | 1134 | 53-374 | 8 | 1 | 8.99/39586.51 | 42.63* | 0.534 | 106.92 | P | 305 (LTKVYRENSPT) |
| 5 | Pgl_GLEAN_10011825 | PglNramp2 | Nramp Metal Transporter Nramp6 | 2 | 367 | 1104 | 111-367 | 5 | 3 | 5.38/40073.23 | 37.26 | 0.266 | 99.4 | P | 130 (LENYGVR) |
| 6 | Pgl_GLEAN_10020447 | PglZIP3 | ZIP Zinc Transporter | 2 | 277 | 834 | 90-263 | 8 | 11 | 7.06/29554.01 | 40.72 | 0.658 | 110.61 | P | 57 (LAHNAINSIG) |
| 7 | Pgl_GLEAN_10002986 | PglYSL1 | OPT Metal Nicotianamine | 3 | 731 | 2196 | 77-702 | 14 | 6 | 8.66/79043.89 | 30.45 | 0.393 | 93 | P | 102 (LTTGIIPS) |
| 8 | Pgl_GLEAN_10002987 | PglYSL2* | OPT Metal Nicotianamine | 3 | 65 | 198 | 1-65 | 1 | 0 | 10.02/7146.53 | 29.92 | 0.628 | 115.54 | EC | 37 (LLKQPFTRQ) |
| 9 | Pgl_GLEAN_10003023 | PglZIP4 | Zinc Transporter | 3 | 551 | 1656 | - | 13 | 4 | 8.20/57481.69 | 34.05 | 0.802 | 115.43 | P | - |
| 10 | Pgl_GLEAN_10003646 | PglYSL3 | OPT Metal Transporter | 3 | 635 | 1908 | 1-619 | 15 | 6 | 9.43/69392.27 | 30.67 | 0.552 | 103.09 | P | - |
| 11 | Pgl_GLEAN_10004539 | PglYSL4 | OPT Metal Nicotianamine | 3 | 664 | 1995 | 1-636 | 11 | 6 | 9.05/72821.53 | 30.69 | 0.499 | 98.46 | P | 11 (LTTGIIPSL) |
| 12 | Pgl_GLEAN_10005017 | PglYSL5 | OPT Iron-phytosiderophore transporter yellow stripe 1 | 3 | 678 | 2037 | 46-662 | 12 | 6 | 9.05/74265.27 | 28.5 | 0.406 | 95.71 | P | - |
| 13 | Pgl_GLEAN_10005018 | PglYSL6 | OPT Metal Nicotianamine | 3 | 543 | 1632 | 13-527 | 11 | 5 | 9.15/59092.25 | 32.55 | 0.481 | 98.42 | P | - |
| 14 | Pgl_GLEAN_10005019 | PglYSL7 | OPT Metal Nicotianamine | 3 | 579 | 1740 | 53-178 | 10 | 5 | 6.20/62270.89 | 30.14 | 0.481 | 103.42 | P | - |
| 15 | Pgl_GLEAN_10008567 | PglYSL8 | OPT Metal Nicotianamine | 3 | 507 | 1524 | 1-196 | 11 | 6 | 9.01/55313.18 | 27.03 | 0.557 | 101.58 | P | - |
| 16 | Pgl_GLEAN_10012759 | PglZIP5 | ZIP Zinc Transporter | 3 | 234 | 705 | 43-234 | 4 | 2 | 6.66/24937.01 | 41.13* | 0.314 | 104.32 | P | 50 (IASILTAGAAG) |
| 17 | Pgl_GLEAN_10031862 | PglYSL9 | OPT Metal Nicotianamine | 3 | 687 | 2064 | 128-674 | 11 | 9 | 9.27/73225.31 | 39.57 | 0.369 | 93.68 | P | 20 (IVLGAAAAAAA) |
| 18 | Pgl_GLEAN_10037548 | PglYSL10 | OPT Metal Nicotianamine transporter YSL6 | 3 | 774 | 2325 | 136-756 | 11 | 8 | 9.17/86370.26 | 27.64 | 0.149 | 97.65 | P | 81 (LDKLEQKEL) |
| 19 | Pgl_GLEAN_10037549 | PglYSL11 | OPT Metal Nicotianamine | 3 | 700 | 2103 | 73-682 | 12 | 8 | 8.44/76309.62 | 29.23 | 0.519 | 105.76 | P | 74 (ILGVLFCLI) |
| 20 | Pgl_GLEAN_10015296 | PglNramp3 | Nramp 6 | 4 | 369 | 1110 | 1-297 | 8 | 3 | 9.11/40416.82 | 27.06 | 0.678 | 122.6 | P/V | 41 (LSLENYGVRK) |
| 21 | Pgl_GLEAN_10019761 | PglZIP6 | Zinc Transporter | 4 | 228 | 687 | 39-214 | 6 | 9 | 5.39/24633.06 | 35.25 | 0.684 | 107.85 | V | - |
| 22 | Pgl_GLEAN_10023965 | PglNramp4 | Metal Transporter Nramp3 isoform X2 | 5 | 443 | 1332 | 68-306 | 8 | 11 | 7.64/47914.23 | 28.79 | 0.585 | 117.31 | Chl | 185 (QYGVRKLEF) |
| 23 | Pgl_GLEAN_10030145 | PglNAS1 | Nicotianamine synthase 2 | 5 | 883 | 2652 | 3-282 | 0 | 0 | 6.00/96605.68 | 37.55 | -0.05 | 97.69 | C | - |
| 24 | Pgl_GLEAN_10034360 | PglZIP7 | Zinc Transporter like | 5 | 276 | 831 | 2-272 | 7 | 3 | 5.83/29119.89 | 28.95 | 0.654 | 108.62 | V | 131 (LCFHSVFE) |
| 25 | Pgl_GLEAN_10001420 | PglZIP8 | Zinc Transporter | 6 | 348 | 1047 | 65-345 | 9 | 0 | 7.69/37487.65 | 41.24* | 0.478 | 101.9 | P | 20 (LLSGLLLCSSQQ) |
| 26 | Pgl_GLEAN_10012010 | PglYSL12 | Metal-nicotianamine transporter YSL18 | 6 | 680 | 2043 | 28-649 | 15 | 5 | 9.19/73552.93 | 33.09 | 0.469 | 100.99 | P | - |
| 27 | Pgl_GLEAN_10000860 | PglNramp5 | Metal Transporter Nramp | 7 | 545 | 1638 | 83-446 | 12 | 12 | 7.14/59099.11 | 42.98* | 0.767 | 132.59 | P | 37 (LLCHFLIQIWFPP) |
| 28 | Pgl_GLEAN_10002291 | PglNramp6 | Metal Transporter Nramp 5 | 7 | 504 | 1515 | 34-397 | 11 | 10 | 8.36/55104.17 | 41.19* | 0.633 | 122.5 | P | 43 (IGLIFALIQSL) |
| 29 | Pgl_GLEAN_10036448 | PglZIP9 | Zinc Transporter | 7 | 186 | 561 | 21-183 | 4 | 2 | 6.82/19309.08 | 19 | 0.953 | 127.37 | V | 39 (LGIVVHSV) |

* PglYSL2 found to be less number of amino acids hence we are not considered as full length gene

V (Vacules), P (Plastid), Chl (chloroplast), EC (Extra Cellular), ZIP(zinc and iron regulated protein), YSL (Yeast Strip Like), Nramp (natural resistance- associated macrophage protein), NAS (nicotianamine synthase), FER (Ferritin like).
