# Supplementary material for: Discovery and validation of candidate genes for grain iron and zinc metabolism in pearl millet [Pennisetum glaucum (L.) R. Br.]
Source: Sci Rep. 2020 Oct 6;10:16562. doi: 10.1038/s41598-020-73241-7 (PMC7538586; doi:10.1038/s41598-020-73241-7)
Supplement: Supplementary file 7 [file 41598_2020_73241_MOESM7_ESM.pptx]

## Slide 1
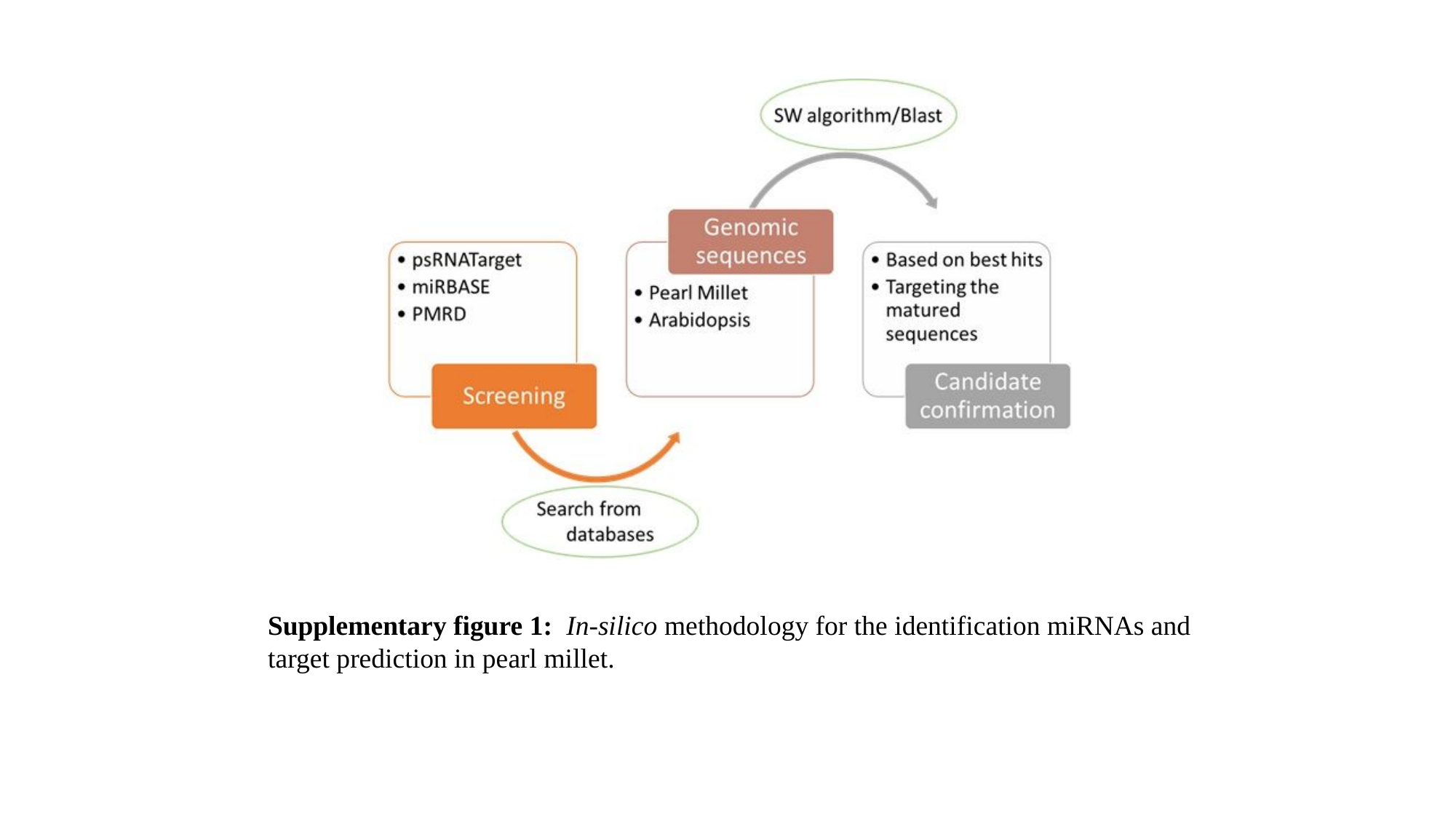

Supplementary figure 1: In-silico methodology for the identification miRNAs and target prediction in pearl millet.
